# A systematic review and meta-synthesis of the impact of low back pain on people’s lives

**DOI:** 10.1186/1471-2474-15-50

**Published:** 2014-02-21

**Authors:** Robert Froud, Sue Patterson, Sandra Eldridge, Clive Seale, Tamar Pincus, Dévan Rajendran, Christian Fossum, Martin Underwood

**Affiliations:** 1 Warwick Clinical Trials Unit, Warwick Medical School, Gibbet Hill Road, Coventry CV4 7AL, UK; 2 Centre for Primary Care and Public Health, Queen Mary University of London, 58 Turner Street, Whitechapel, London E1 2AB, UK; 3 Metro North Mental Health, Royal Brisbane and Womens’ Hospital, Brisbane, Queensland 4029, Australia; 4 School of Social Sciences, Brunel University, Uxbridge UB8 3PH, UK; 5 Department of Psychology, Royal Holloway, University of London, Egham, Surrey TW20 0EX, UK; 6 University College of Health Sciences, Campus Kristiania, Prinsens gate 7-9, 0153 Oslo, Norway

**Keywords:** Outcome measurement, Outcome measure development, Low back pain, Qualitative synthesis, Social factors, Population-based interventions

## Abstract

**Background:**

Low back pain (LBP) is a common and costly problem that many interpret within a biopsychosocial model. There is renewed concern that core-sets of outcome measures do not capture what is important. To inform debate about the coverage of back pain outcome measure core-sets, and to suggest areas worthy of exploration within healthcare consultations, we have synthesised the qualitative literature on the impact of low back pain on people’s lives.

**Methods:**

Two reviewers searched CINAHL, Embase, PsycINFO, PEDro, and Medline, identifying qualitative studies of people’s experiences of non-specific LBP. Abstracted data were thematic coded and synthesised using a meta-ethnographic, and a meta-narrative approach.

**Results:**

We included 49 papers describing 42 studies. Patients are concerned with engagement in meaningful activities; but they also want to be believed and have their experiences and identity, as someone ‘doing battle’ with pain, validated. Patients seek diagnosis, treatment, and cure, but also reassurance of the absence of pathology. Some struggle to meet social expectations and obligations. When these are achieved, the credibility of their pain/disability claims can be jeopardised. Others withdraw, fearful of disapproval, or unable or unwilling to accommodate social demands. Patients generally seek to regain their pre-pain levels of health, and physical and emotional stability. After time, this can be perceived to become unrealistic and some adjust their expectations accordingly.

**Conclusions:**

The social component of the biopsychosocial model is not well represented in current core-sets of outcome measures. Clinicians should appreciate that the broader impact of low back pain includes social factors; this may be crucial to improving patients’ experiences of health care. Researchers should consider social factors to help develop a portfolio of more relevant outcome measures.

## Background

Low back pain (LBP) was the biggest contributor globally to Years Lived with Disability (YLDs) in the most recent Global Burden of Disease Study, dropping to just sixth as the biggest contributor to DALYs (Years of life lost + YLDs) [1,2]. LBP is the most common form of chronic pain, and in the UK it is a National Health Service research priority [3]. The estimated cost to the UK economy was over £6.6 billion, in 1998 [4]. Approximately 4% of the UK population take time off-work because of LBP, equating to around 90 million working days lost, and between eight and 12 million GP consultations per year [5]. Whilst 90% of patients who consult GPs for LBP in the UK cease consulting within three months, most are still experiencing LBP and related disability one year after consultation [6]. This suggests that either those affected feel that there is limited help available, or that for another reason consultation is not worthwhile.

Notwithstanding the biopsychosocial model playing a driving role in LBP research since the model was proposed, not all components of the trinity are well-represented in back-specific sets of core outcome measures [7-9]. Pain and disability (function) are the most common constructs measured in trials of back pain treatments [10,11]. This has received criticism, particularly in terms of missing relevant domains and time-frames, and the breadth of coverage of measurement [12-14]. The IMMPACT recommendations, which pertain to chronic pain generally rather than back pain *per se*, suggest ‘emotional functioning’ is measured in addition to pain and physical function, global improvement, disposition, symptoms, and adverse events [15]. Back-specific core-sets include pain, function, and disability, satisfaction, general well-being, and mental health [9,16]. The divergence between the aetiological model and our measurement of outcome deserves consideration.

The development of ‘next generation’ outcomes for research and clinical use needs to take a broader perspective, with emphasis on patient-importance [17]. To help inform this process, we report the impact of non-specific LBP on patients’ lives, in a meta-synthesis of qualitative studies. Several methods have been advanced for combining qualitative research, from meta-narrative, to rigorous coding, and translation of themes, and there is not yet consensus on best practice [18-20]. We used a systematic review to identify qualitative studies of patients’ experiences of chronic non-specific LBP and then a meta-narrative approach and a meta-ethnographic approach to explore the impact chronic non-specific LBP had on people’s lives, and whether these two approaches led to any material interpretive differences.

## Methods

### Databases searched and inclusion and exclusion criteria

To identify qualitative studies of patients’ experiences of chronic (*i.e.* ≥ 12 weeks) non-specific LBP, published in English peer-reviewed journals, from database inception until July, 2011, we searched CINAHL, Embase, PsycINFO, PEDro, and Medline [21]. Whilst PEDro primarily indexes clinical trials, systematic reviews, and guidelines, our scoping searches revealed it to index a nested qualitative study within a back pain trial, hence our inclusion of this database. We included all studies in which conclusions were based on qualitative data collected during face-to-face interviews or focus groups, reporting patients’ experiences of chronic non-specific LBP. We excluded studies solely reporting on experiences of trial participation.

### Search strategy

We used terms that were developed from the Cochrane back review search strategy, scoping searches, and team discussion [22]. Our search strategy is detailed in full online (Additional file 1: Table S1). Briefly, it included variations on the following terms: ‘*low back pain, backache, lumbago, grounded theory, interview, focus group, phenomenology, action research, ethnographic, and epistemology*’. Two reviewers (RF & SP) independently searched titles and abstracts, and full texts where necessary, to agree on included studies. Disagreements were resolved by an arbitrator (MU).

### Appraisal of included papers

Two of four researchers (RF, SP, DR, or CF) independently assessed the reporting of each included study against the Consolidated Criteria for Reporting Qualitative Research (COREQ) framework [23,24]. Disagreements were resolved by arbitration by one of the remaining two researchers. RF and DR also abstracted data on described analytical approaches, individual study aims, and findings, for provision as an online resource.

### Data extraction and analysis

Regarding the most appropriate methodology for qualitative synthesis. We modified an approach described by Britten and colleagues, using Marston and King’s approach to coding [18,19]. Britten developed a seven-step approach originally described by Noblit and Hare [25]. The first three steps encompass 1) ‘getting started’, *i.e.* identifying the research area of interest, what has previously been done, gaps in research, and forming a specific research question, *e.g. What is the impact of non-specific low back pain on patients’ lives?* 2) ‘deciding what is relevant’, in terms of choosing material that is broad enough to enable the research question to be addressed, whilst not too disparate to raise questions regarding commensurability. In our case, we focused on any qualitative research featuring face-to-face discussions about the experience of non-specific low back pain; and then 3) ‘careful reading and re-reading’ of all included studies. The fourth step involves ‘determining how the studies relate to each other’, which was done by considering relationships between the concepts arising from the included papers; for example, work-based stigma being perceived by participants to be associated with the ‘invisibility’ of the condition, or a lack of credible diagnosis. A grid of concepts is formed during the fifth step and studies are ‘translated into one another’, which involves paraphrasing concepts in the grid cells – *e.g.* stigma, depression, trouble sleeping, etc. We modified the fourth and fifth steps using Marston and King’s approach, which permits concepts and themes to be rigorously and systematically considered following coding, and the synthesis, once expressed, to be supported by typifying quotes and descriptions of individual study participants’ responses (first-order constructs). For example, consider an original (first-order) quote presented in an included study under the theme of ‘confrontation’, stated “*I remember the look in my managers eyes when I spoke of my back pain – I knew he didn’t believe me*”. We might code this under a theme with the same name, and/or re-code it under different themes in-line with our research question, for example ‘stigma’, ‘deligitimisation’, or ‘perceived disbelief’. Our coded themes were assigned to nodes, which facilitated their arrangement to form a framework; for example, a ‘relationships’ theme might comprise sub-themes of nodes containing coding for family relationships, social relationships, sexual relationships, and so on. We also separately coded authors’ interpretations of the impact of LBP (second-order constructs), when these were reported [26]. We had access only to data presented by authors in the included study reports, and not to any raw data from these included studies. We did not seek validation of our coding from the original authors of our included studies.

RF thematically coded the first-order constructs within a thematic framework that was developed by RF, SP, and TP, following a preliminary analysis of selected studies. The coding framework was modified by RF as remaining studies were reviewed, with these modifications agreed with SP and TP. RF coded second-order constructs independently of the thematic framework. Nvivo 9 (QSR International, Victoria) was used to manage these data and their coding.

A second reviewer (SP) independently reviewed all included studies and analysed data using a meta-narrative approach described by Greenhalgh and colleagues, which involved identifying the key dimensions of the research problem, taking each dimension in turn and giving a narrative account of its contributions, before summarising the overall messages from the research literature [20]. The results of both processes were then compared and informed the sixth step of synthesis as described by Britten and colleagues, ‘synthesising translations’ [18]. This involved considering each of the concepts and second-order constructs in turn, the meta-narrative review, and whether these were refutational or reciprocal [18]. For example, if an interpretation in one study is that people with back pain strongly depend on close relationships for emotional support, yet an interpretation in another study is that people in pain seek solitude, distancing themselves from close relationships, one might consider these interpretations refutational. However, in the case a third study suggests that people with back pain depend on close relationships for emotional support generally, but isolate themselves from others during periods of intense pain, one might reason these interpretations are reciprocal, and go on to form an argument that back pain suffers generally depend on close relationships but seek solitude during episodes of relatively intense pain. RF, SP, MU, TP, CS, and SE all contributed to the synthesis following discussion and deliberation of coded constructs and the meta-narrative description. The seventh and final step is the expression of the synthesis, which we do in this paper. We describe first and second-order themes, as well as our third-order interpretation.

## Results

**Figure 1:**
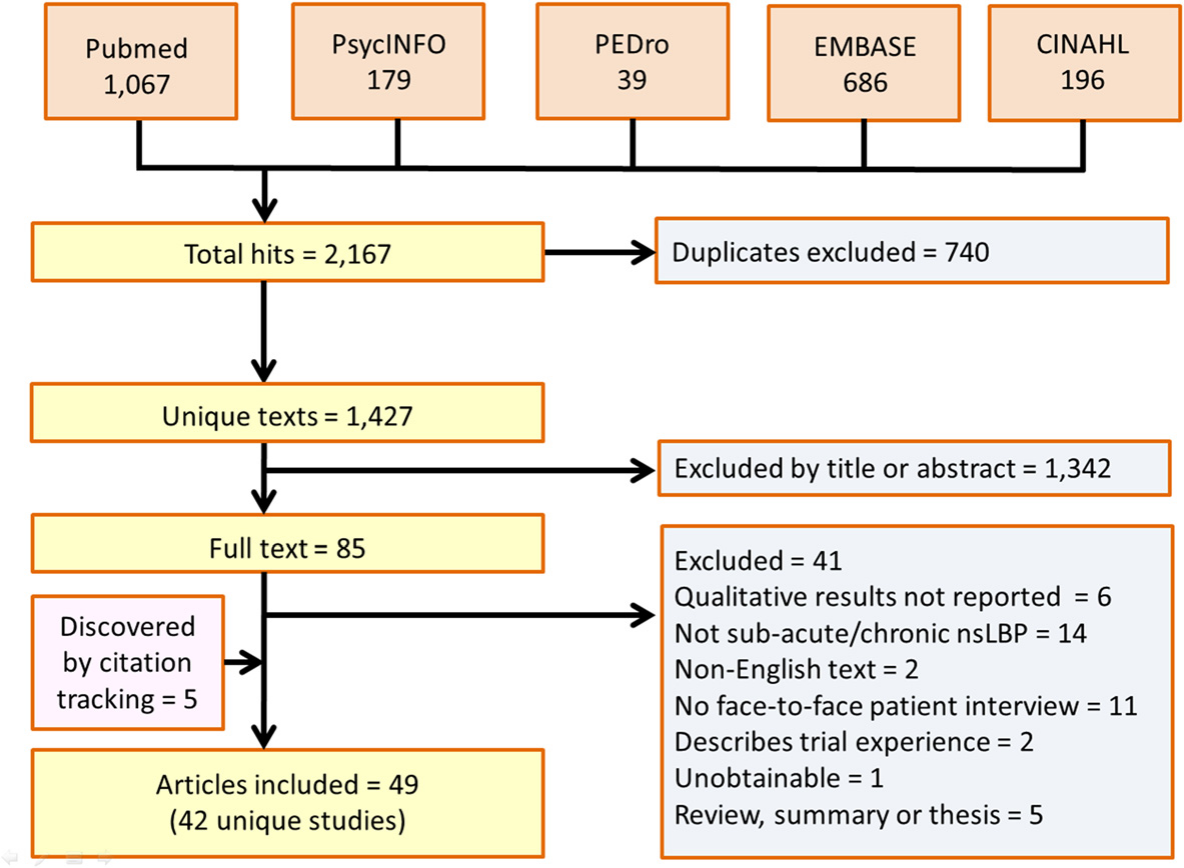
Flow chart showing study identification, and numbers and types of excluded studies, and numbers of included studies.

From 1,427 unique citations, we excluded 1,342 on the basis of titles and abstracts (Figure 1). After examining full-texts for the remaining 85 citations, we excluded 41 studies. We identified five further papers from citation tracking [27-31], finally including 49 papers describing 42 unique studies [12,13,27-73]. Characteristics of the included studies are shown in Table 1 and additional information including the main findings from each of our included studies can be found in our Additional file 2: Table S2. The completeness of reporting of the included studies was variable as judged by the COREQ framework (Additional file 3).

**Table 1:** Characteristics of included studies

**Lead author & year**	**Country**	**Sample size**	**% Female**	**Central age (mean/median)**	**Aim**	**Setting**
Allegretti, 2010 [32]	USA	23	48	45	To explore patient and physician interviews and improve communication	Family care centre
Benjaminsson, 2007 [33]	Sweden	17	59	36	To explore how patients respond to recurrence of pain	Physiotherapy clinic
Borkan, 1995 [34]	Israel	66	65	39.5	To explore patients’ perceptions and experiences	Family practice, clinic, or home
Bowman, 1991 [35]	USA	15	40	ND	To investigate the meaning of chronic LBP	ND
Bowman, 1994a [36]	USA	15	40	ND	To describe life with LBP	Pain management centres
Bowman, 1994b [37]	USA	15	40	ND	To examine the reaction of individuals to chronic LBP	ND
Busch, 2005 [38]	Sweden	22	68	41	To examine the development of pain related appraisals, coping and well behaviours, as well as to investigate how these processes affect one another during the course of LBP	Private clinic room
Campbell, 2007 [39]	UK	16	ND	ND	To examine expectations for pain treatment and outcome and to determine whether they are influential in maintaining health service consumption	ND
Chew, 1997 [40]	UK	20	82	ND	To explore how sufferers of LBP describe their pain and its impact on their lives	ND
Cook, 2000 [41]	ND	7	57	42.3	To gain an in-depth understanding of individual patients’ experiences of LBP and active rehabilitation	Home, or physiotherapy clinic
Coole, 2010 [31]	UK	25	52	44.7	To explore the experiences of employed people with back pain regarding the help they have received from GPs	Home, workplace, or local clinic
Coole, 2010 [42]	UK	25	52	44.7	To explore the individual experiences and perceptions of patients awaiting rehabilitation who were concerned about their ability to work because of persisting, or recurrent, low back pain	Home, workplace, or local clinic
Corbett, 2007 [43]	UK	37	59	ND	To explore the struggle between hope and despair through consideration of six people’s narratives about their experiences of chronic LBP	Home, or research centre
Crowe, 2010 [44]	New Zealand	64	48	55.1	To investigate experiences of the impact of LBP	ND
Dean, 2010 [47]	New Zealand	33	18	47.7	To explore and document the experiences of NZ farm workers who continue to work despite their LBP	‘Place of convenience to the participant’
Hooper, 2005 [49]	UK	5	50	ND	To provide opportunities to reflect on clinical practice and on the role of informal carers within the provision of health care for the back pain patient	ND, although it is clear that a participant with back pain has been interviewed with his wife (also his expert carer)
Holloway, 2007 [48]	UK	18	50	53	To explore and conceptualise the experiences of people of working age who seek help from pain clinics for LBP	Patients’ homes
Hush, 2009 [12]	Australia	36	42	41	To explore patients’ perceptions of recovery from LBP	Meeting room at University of Sydney
Hush, 2010 [13]	Australia	36	42	41.6	To explore whether NRS/RMDQ capture meaningful changes	ND
Keen, 1999 [27]	UK	27	37	ND	To explore the association influence changes in physical activity and the way individuals perceive and behave with their LBP and the impact of this on physical activity	Homes of participants, and office of PI
Layzell, 2001 [50]	UK	12	50	ND	To explore how back pain affects sufferers’ lives	ND
Liddle, 2007 [51]	New Zealand	28	78	ND	To explore experiences, opinion, and treatment expectations of LBP to identify what treatment components are valued	Private room in university
May, 2000 [52]	UK	12	50	ND	To explore ways persons with long standing chronic LBP respond to medical doubt about the presence of organic pathology	ND
Morris, 2004 [53]	UK	6	50	ND	Patients’ experiences of attending a back rehabilitation programme were examined	Participant choice of home, quiet room in hospital, or clinic
Ong, 2003 [28]	UK	6	50	ND	To describe course of LBP over 12 months	ND
Ong, 2004 [54]	UK	16	38	ND	To explore how people report LBP to clinicians	Patients’ homes
Ong, 2006 [29]	UK	2	100	ND	To explore the role of concordance in therapeutic relationships through directly comparing patients’ and clinicians’ accounts of the diagnosis and impact of LBP	Patients’ homes
Osborn, 1998 [55]	UK	9	100	ND	To explore the sufferer’s personal experiences of their pain	ND
Osborn, 2006 [56]	UK	6	40	44	To explore and articulate the meanings and themes that make up the personal experience of the body when in pain	ND
Reid, 2004 [57]	UK	50	54	ND	To explore the perceived health needs of chronic LBP patients	Homes and clinics
Skelton, 1996 [58]	UK	52	50	41	To elicit the views of patients concerning LBP and its management in general practice	ND
Slade, 2009 [61]	Australia	18	50	51.2	To determine participant-experience of exercise programmes for non-specific LBP	ND
Slade, 2009 [59]	Australia	18	50	51	To determine what factors are important for patients to engage in exercise programmes	ND
Slade, 2009 [60]	Australia	18	50	51	To investigate and summarise participant experience of exercise programmes for non-specific LBP and the effects of these experiences on exercise participation and engagement	ND
Sloots, 2010 [62]	Netherlands	23	52	40	To explore which factors led to drop-out in patients of Turkish and Moroccan origin with chronic nonspecific LBP who participated in a rehabilitation programme	Participant choice of home or clinic
Smith, 2007 [63]	UK	6	33	44	To explore how chronic benign low back pain may have a serious debilitating impact on the sufferer’s sense of self	ND
Snelgrove, 2009 [64]	UK	10	70	ND	To understand the meaning of LBP for participants with longstanding history of chronic pain	Patients’ homes
de Souza, 2007 [45]	UK	11	55	49.3	To explore and describe the physical consequences of living day-to-day with LBP and to document insider accounts of how the pain impacts daily activities	Participants’ homes
de Souza, 2011 [46]	UK	11	55	49.3	To explore interactions and relationships within the family and the workplace from the perspective of the person with chronic spinal pain	Participants’ homes
Sokunbi, 2010 [65]	UK	9	67	46.6	To explore the experiences of a sample of individuals with chronic LBP who participated in an RCT of exercises	Private room in university
Strong, 1995 [67]	New Zealand	19	58	53.7	To explore coping strategies	ND
Strong, 1994 [66]	Australia	8	50	54.4	To explore relevant dimensions of pain	Private room in Brisbane Royal Hospital
Tarasuk, 1995 [30]	Canada	15	33	ND	To learn about individuals’ experiences and perspectives of longer term ramifications of LBP	ND
Tavafian, 2008 [68]	Iran	24	100	42.9	To explore Iranian womens’ beliefs about causation	ND
Tveito, 2010 [69]	USA	15	33	ND	To address legitimacy concerns in the workplace, particularly those relating to workers’ perceptions of reactions of employers, supervisors, and co-workers	Quiet office
Young, 2011 [73]	Canada	31	45	ND	To determine the meaning participants associated with the term ‘recurrence’	Public library in Vancouver
Wade, 2003 [70]	South Africa	3	100	ND	To provide a description of the life-world of people with chronic low back pain	ND
Walker, 1999 [71]	UK	20	40	ND	ND	Participants’ homes
Walker, 2006 [72]	UK	20	40	ND	To provide a more detailed understanding of the lived experience of chronic back pain prior to seeking help from pain clinics	Participants’ homes

ND = No datum(a).

### First-order themes

Five major first order themes were identified from participant-level data. Unless otherwise indicated, findings were not associated with study country or setting.

#### ***Theme 1: activities***

Loss of function undermined the ability to perform activities. The greatest impact was to domestic chores, valued recreational activities, and to planning ahead. Sub-themes included domestic issues, difficulty with leisure activities, rest and sleep, unpredictability surrounding the planning of future activities, and needs associated with activities employed for coping with pain.

Participants described difficulties with gardening, housework, and shopping (*See quotes 1, 2*; Table 2) [13,50,57,68]. Sleep, leisure activities, and outlets for stress that participants had previously enjoyed were often no longer available (*Quote 3*) [45,57]. The inability to predict the onset of pain led to anticipation of pain that compromised the ability to plan ahead, leading to a convoluted mental decision-making processes surrounding participation [35,38,67]. Many emphasised the need for vigilance and a need to take painkillers in order to accommodate function and to enable activity and participation [44,64,67].

**Table 2:** Selected quotations from included studies

**Theme; subtheme**	**Quote number**	**Quote**
Activities; domestic	1	“*As soon as my back goes: that’s it, I don’t mow my lawn for a couple of months.*” (Mick,40) [13]
	2	“*Things like [cleaning the] bathroom and shower and stuff, because you have to get right in and you’re bending over when you’re scrubbing*.” (Angela, 35) [13]
Activities; Leisure, rest, and sleep	3	“*I would go in the garden and do a bit of this and a bit of that, you know, but now I just don’t bother. … I used to go and play golf… to relax, and things like that.”* (Subject 1) [46]
Relationships	4	“*The worst thing about this pain is that you are in pain, yet everybody else suffers with you*.” (Patient 1) [46]
Relationships; damage and isolation	5	“I’ve given up on holidays because it spoils it for everyone else” (Patient 4) [39]
	6	“Your wife says “come on, get your act together” and that makes you feel bloody terrible.” (Patient 13) [39]
Relationships; family and cohabitation	7	“My oldest son, a four year old, says, “What is it Daddy, you used to hold me in your arms, why don’t you now?” (Anon.) [34]
	8	“My wife even turned on me, thinking it was all put on. She came into the bedroom one morning to find me flat on the floor, unable to move, and she naturally assumed that I was putting it on. From that point on I’ve just lived on my own.” (Colin, 46) [72]
Relationships; sex	9	“Sex, sex is very important. It’s very important” (Patient 4) [46]
	10	“I mean you don’t look ill, you’re not flat on your back, so you know, is it an excuse, ‘oh I’ve got a headache’, do you know what I mean?” (Ruth) [55]
Relationships; social	11	“You go out to a restaurant, halfway through a meal, because you’ve been sat for too long, I have to get up and go for a walk.” (Anon.) [41]
	12	“…we won’t go anywhere now because of that [being boring with little to talk about except pain]. I get too embarrassed and I just hate being in company and you always get onto that subject [pain]. And if you’re out for a social evening the last thing people want to hear is what your misery, so I just, that’s why we don’t go out often.” (Becky) [55]
	13	I don’t go out, I don’t answer the phone, I live at the back of the house and I dread it when the postman comes. … I don’t know what to say, or anything, I just feel embarrassed. You just think ‘what do they think of me?’” (Kevin) [63]
Work; anxiety	14	“My reading is poor, I can’t spell for jack… it’s like I’m in a no-win situation … All my work comes physical” (Patient 12) [32]
Work; off-sick	15	“I don’t look sick, I don’t limp, I don’t have a cane, I’m not in a wheelchair, I don’t look terrible … I look good. So [the people I work with] could have the perception that she’s not really sick, she’s just taking days off” (Participant 14) [30]
Work; financial	16	“I didn’t know what to do … they [doctors] said ‘there’s nothing there, there’s nothing there whatever’… so I was scared of chiropractor, and of course I couldn’t afford it either, so there was massage therapy - I couldn’t afford that either. Which one is the cheapest? Acupuncture! So I looked through the Yellow Pages and there was one and I said I’ll give him a call.” (Participant FG5) [73]
	17	“I can’t go off-sick. I can’t afford to go on half-pay [incapacity]. So … so that’s a real dilemma and then I think: God, I have to work until I’m 65! I’ve got a mortgage to pay. How am I going to cope? … You start thinking: what if it never goes, right? What if it gets worse? What am I going to do?” (Anon.) [43]
Stigma; deligitimisation	18	“I remember at my sickness interview - you can see the disbelief in the manager’s eyes, and I’m thinking OK well …” (male, aged 37) [42]
Stigma; diagnosis	19	“..but you can’t see pain, so they don’t know do they? So they automatically assume that there’s nowt wrong with you” (Alice) [55]
	20	“I just don’t appreciate them trying to tell me that the pain is in my head. You know, in so many words they tell me the pain is in my head and I have feelings in my back … like I say they feel it’s in my head or I’m fronting my back pain.” (Anon.) [37]
	21	“…it always seems sub-consciously that malingering thing, you can’t put your finger what it is, you haven’t got a broken leg or … You have to have stitches to show for it or something…” (Carolyn) [61]
	22	“It’s frustrating sometimes when [going to] a doctor -- yes they’ve studied it, but they haven’t lived it” (Participant FG2) [73]
Stigma; meeting expectations	23	“A very arrogant [doctor] sat me down and said `What the bloody hell do you expect me to do if you are still working?’ And because I was still working, obviously my back wasn’t that bad. But it was.” (Sufferer 1) [28]
	24	“When I’m good, I’m really good, so you walk around and people say `Why did you retire?’ I’ve had some people sort of either directly or imply `look you’re up and walking around, what’s your problem?” (Alex, 57) [61]
Changing outlook; quest for diagnosis	25	“I found out since that it’s not been diagnosed correctly. They’ve been giving me the wrong exercises for somebody with what I’ve got now. For 10 years I’ve been doing exercises according to this type of pain, when it’s been aggravating the other thing that was never diagnosed, it was always there but they never looked at it.” (Jean) [61]
Changing outlook; psycho-emotional	26	“I mean, I’ve had days and weeks where I’ve just got depressed over it, and I think, well, I can’t be bothered, there’s no point, it’s not getting better.” (Anon.) [41]
	27	“Oh aye, aye, I’m down in the dumps most of the time as [wife] knows. If it wasn’t for the missus I’d be bloomin’ terrible I think.” (Will) [64]
	28	“I’d love that [being alone on a desert island] … but to be away from people and not to have to be something else you’re not, that would be bliss. … I’d still be a miserable old git but it wouldn’t matter, its only when other people come around that it matters.” (Tony) [63]
	29	“Oh yeah, its in two parts, the old good bit, and the pain bit, which has gone wrong. … One bit works, the other doesn’t, like a section has gone wrong, when it’s bad and I can’t move properly, it’s like it’s not part of me, it won’t obey.” (Lynette) [56]
	30	“I felt like a wasp with a very tiny waist. Just imagine! Such a waist may snap anytime! It was horrible, I just couldn’t move! I didn’t think I’d make it.” (Anon.) [38]
Changing outlook; adaptation and acceptance	31	“I would like to take medical retirement … it would be nicer to actually say to people ‘I’m retired’ rather than ‘I’m off sick’ (Reg) [48]
	32	“After a bad night I can’t settle, but the only way I find if after you’ve taken the pain killers, and the pain is still there, is to actually slide off the chair and kneel facing the chair, taking all the weight on my knees” (Anon.) [64]

#### ***Theme 2: relationships***

Participants described damaged relationships; most notably with those closest to them (*Quote 4*). Sub-themes included descriptions of damage, feelings of isolation, family and cohabitation difficulties, issues surrounding sexual relations, and issues surrounding social interaction.

Participants expressed a paradoxical need/desire for support from those closest to them, whilst simultaneously wanting to avoid those close to them whilst in pain [37]. Some avoided family activities because they feared spoiling the experience for loved-ones (*Quote 5*). Others felt unsupported (*Quote 6*) [39,46]. Some engaged in activities they thought likely to exacerbate their symptoms, simply to maintain relations, emphasising the price of this could be a loss of credibility, since participation could be perceived as evidence that there is actually nothing wrong [48]. Some described a high degree of dependence on others [40]. Activity limitations were considered to have negative implications for relationships, especially in terms of interacting with children or grandchildren (*Quote 7*) [63,66]. In some cases, marital relationships suffered such that cohabitation became unviable (*Quote 8*) [72]. Being able to maintain valued sexual relationships was considered very important by some; the absence of sexual activity due to LBP was associated with perception of a damaged relationship (*Quotes 9, 10*) [34,46]. A cognitive dissonance was evident in accounts of social interaction. On the one hand, participants described themselves as social, or formerly social, and wanted to be able to go out with friends. On the other hand, they recounted uncomfortable feelings associated with social activities; including being worried about how they were seen by others, fear of having to sit in pain for protracted periods of time, and fear of spoiling events for others [41,63,68]. Such fears led to social withdrawal and isolation (*Quotes 11, 12, 13*) [55]. One study stands out in terms showing a largely positive effect of a supportive relationship; however it is notable that the context this study is atypical in that it included a joint interview with the back pain sufferer and his wife, in addition to separate interviews with health care providers [49].

#### ***Theme 3: work***

Participants emphasised the need to modify work tasks, the fear associated with losing a job, and the interpersonal challenges that arise following the disbelief of co-workers. Sub-themes identified included anxiety, modifications to work-related activities, interpersonal relationships, time off-sick, and financial worries.

Many worried about loss of employment (*Quote 14*) [32,34]. Younger participants tended to perceive back pain as more threatening to their careers; whereas older workers, or those closer to retirement appeared to find it easier to ask for help [38,42,69]. Some did not disclose their back problem to their employers, fearing the employer may be unwilling to accommodate their needs, or might terminate their employment [69]. Others, following dismissal from work or transfer, felt that absence or disclosure of back pain had been responsible [30,71]. Modifying tasks, where possible, was thought to facilitate function [35]. Allowing sufficient time to recover was seen as one such important modification; however, it was emphasised that this sometimes resulted in time off-work [42,73]. One participant described using holiday for recovery rather than taking time off-work, which he believed would pose a risk to his job [72].

Many suggested that co-workers did not regard their back pain claims as legitimate. Participants battled to be believed, making efforts to perform tasks in spite of pain. Seen as a sign of competence, there was a worry that this could serve to further fuel delegitimisation of their pain in the eyes of co-workers [69]. Some began to question their worth as an employee [69]. Participants not eligible for sick pay, often described not being able to afford to take the time off. Others pointed out reduced pay could be insufficient, and some worried about job security or the stigma from co-workers that could result from taking sick leave (*Quote 15*) [30,43,46]. Many were concerned about the ability to maintain bill payments [35,42-44]; and some were concerned about the cost of therapy (*Quotes 16, 17*) [73]. One participant reported losing his home as an indirect result of having back pain, and others described descents into poverty [72].

#### ***Theme 4: stigma***

Concerns surrounding legitimacy, credibility, and validation, permeate accounts of life with low back pain. These included not being believed by family, friends, employers, and health care providers [34,37]. Patients sought to employ various strategies to establish themselves as credible [34,48]. Identified sub-themes comprised delegitimisation, a frustrating lack of diagnosis, and the ability to meet expectations.

Delegitimisation was experienced both directly in relation to claims to be in pain, and indirectly in relation to consequences of back pain (*Quote 18*) [30]. The delegitimisation was thought to arise from the absence of identifiable pathology and hence of there being no adequate or acceptable diagnosis [43,50]. Participants were frustrated at being left without sufficient explanations for their inability to perform activities [34,37,61]. Some proposed that it was because there was nothing visibly wrong and because they had no diagnosis that they struggled to be believed (*Quotes 19, 20, 21, 22*) [30,55,64]. In cases where diagnoses had been given, participants felt legitimised [30,54,61]. Some described the experience of stigma as so powerful that they themselves began to actively question whether their claim to illness might be unwarranted [57,69]. Participants with variable pain who had claimed not to be able to perform certain activities explained that on good days they might be found working at home in their gardens, or in one example, renovating apartments [34]. Such apparent contradictions served to endorse the perceived view of others that the pain is not real [28]. Some responded by amplifying symptoms in an effort to try to redress the balance; others withdrew from family, friends, and physicians, rather than face stigmatisation (*Quotes 23, 24*) [34].

#### ***Theme 5: changing outlook***

Some participants described changing outlooks after accepting they are unlikely to get an acceptable diagnosis, and that they might not improve in-line with initial expectations. Those managing to adapt and change their outlook, appeared more able to cope [67,70]. Identified sub-themes comprised searching for a diagnosis, psychological and emotional experiences, and adaptation and acceptance.

Second diagnoses that differed from initial diagnoses gave rise to anger and confusion, especially if previous explanations had implied a psychosomatic origin [28,61]. Those who had received a diagnosis appeared more empowered to accept their back pain, and to adapt to the predicament more readily; especially if the diagnosis was in the form of radiographic evidence [28,61]. Some doubted the validity of diagnosis (*Quote 25*) [58].

Coded entries for psychological and emotional descriptions were diverse, including experiences of anger, depression, determination, embarrassment, fear of pathology, feeling imprisoned, feelings of inadequacy, frustration, hopelessness or despair, identity threats, insomnia, irritability, isolation, kinesophobia, mood swings, self-loathing, shame, and uselessness. Depression and feelings of hopelessness were commonly described, with few reports of participants considering suicide (*Quote 26, 27, 28*) [50,66,69,70]. Participants feared losing control over their lives, and an uncertain future [50]. Some described changing perceptions of their back (*Quotes 29, 30*) [56,64]. The act of acceptance was felt to be important [50]. Numerous, varied, and often specific practical adaptations were described by participants who had accepted their back pain may not ever go, which included ‘listening’ to their back, avoiding certain situations or tasks, adopting certain postures, doing certain activities, relying on faith/positive thoughts, prioritising their back, or portraying themselves differently to others (*Quote 31, 32*) [37,38,54,58,67,73]. Some patients suggested that respite from activities when needed could be helpful [73].

### Second-order (author) interpretations

Authors’ interpretations emphasised that the impact of back pain was varied, extending beyond functional considerations, and pervading many aspects of patients’ lives. In the worst cases, low back pain dominated participants’ lives with life-changing psychological and social consequences [34]. Particular emphasis was placed on the detrimental effect of stigma and on the events and pathways from which stigma may be generated and experienced [48]. Experiences were thought to affect participants’ self-perception and view of their future, with patients experiencing cyclic journeys through hope and despair [43]. Unpredictability and lack of control were often cited [74], as was the strain under which relationships were placed [46]. Authors suggested that participants may focus on the amelioration of pain in the belief that once this is achieved, normal relationships can be resumed [39]. Authors also emphasised financial concerns, difficulties with work activities and co-workers, and the desire to return to a pre-diseased state [63,69].

### Third-order interpretation (meta-synthesis)

Following examination of second-order interpretations, it was apparent that the studies were not refutations of each other, but described facets of a complex phenomenon. The different approaches taken by the two reviewers converged, yielding similar constructs, and we formed the following line-of-argument describing our interpretation of the literature.

People with low back pain seek to regain their pre-pain healthy, and emotionally robust state. They desire not only diagnoses, treatment and cure, but simultaneously reassurance of the absence of pathology. Practically, although sufferers are often chiefly concerned with (re)engagement in meaningful activities, and attenuation of symptoms, the more experientially-focused literature suggests that the impact of back pain is pervasive, with life-changing effects. These include the inability (or perceived inability) to work; damage to relationships; changing social roles and identity; psycho-emotional problems (especially depression); and worries about the future. Permeating the literature was a sense that patients want to be believed and have validated their experiences and identity as someone doing ‘battle’ with pain. Some may desire to enact the sick role, showing that they are actively engaging with condition and seeking support to return to being a functional member of society. However, in the absence of an acceptable diagnosis, sufferers may feel that they should not adopt the sick role.

Some struggle, but manage to meet others’ expectations, paradoxically undermining the credibility of their pain/disability claims. Others withdraw, fearful of disapprobation and unable or unwilling to accommodate social demands. Over time, some participants who do not find effective therapy begin to accept their low back pain and develop a variety of specific coping mechanisms.

## Discussion

Whilst back pain is not itself life-threatening, it does threaten quality of life. In the absence of diagnosis and effective treatment, complex enmeshment and interactions can ensue between chronic LBP, identity, and social roles, having a diverse and pervasive impact of the condition with life-changing psychological and social consequences. There is little in the data to suggest that individual characteristics, country, or study setting are associated with differences in described impact of LBP. There is some suggestion that age is a factor in determining the impact of perceived threat to career, and one setting in which a back pain sufferer and his wife were interviewed together, described a more supportive relationship than was otherwise typical.

The back-specific core sets of outcome measures recommended by Deyo *et al.* in 1998, and later updated by Bombardier *et al.* in 2000, recommend measurement in the domains of pain, function, well-being, disability, and work disability [8,9]. WHO made back-specific recommendations to measure pain, disability, and depression, in 1999 [75]. International Classifications of functioning (ICF) categories were later proposed, with a core-set of 78 (comprehensive) or 35 (brief) categories being recommended for LBP in 2004 [76]. The brief set, intended in particular for use in clinical studies, has been criticised for incomplete coverage [14,76]. Accepting that both the aetiology and management of LBP fits a biopsychosocial model, and if it follows that outcome measurement coverage should be associated with this trinity, then the recommended coverage may be incomplete. Whilst the bio- component is well-represented within recommendations, psychological factors are less well represented, and the social factors identified by this review are not represented at all in recommendations; excluding the comprehensive ICF core-set, which is likely to be too large to be of practical use in clinical studies. Deyo originally suggested that disability (in parentheses ‘social role’) be measured using the number of days off-work, reduced activities, or bed rest. The domain was later renamed ‘work disability’ in the Bombardier update, which with a shifted focus, it was suggested should be measured using the number of days off work, the number of days of cut-down work, and the time for return-to-work [9,16].

We have found that social factors can be central to the concerns of those with LBP and could be drivers for more costly (to both the individual and to society) and complex sequelae, such as depression. The lack of impetus to measure social factors could be indicative of a more general failure to recognise the role or influence of society in the management of chronic pain conditions. Geoffrey Rose discussed the merits of adopting the strategy of treating the sick population rather than the sick individual [77]; yet so far, population targeted interventions have hitherto been confined to only more obvious public health concerns such as heart disease, smoking, and obesity. The more lateral-thinking future LBP clinical trialist might be tempted to develop a back pain intervention that is aimed at changing population attitudes to back pain. In order for the word ‘illness’ to become ‘wellness’ the ‘I’ must be changed for ‘We’ – by useful coincidence, such rhetoric may serve as an allegory for the sociological changes that may be needed in order to shift the whole population pain distribution to the left, reducing the burden, and in so doing helping sick individuals. One example approach might be the use of media-based interventions, similar to that which have been used in the UK since 2007 to change attitudes and reduce the stigma associated with mental health conditions (http://www.time-to-change.org.uk/about-us/about-our-campaign/start-your-conversation-2013), using a cluster trial design where randomisation is performed by broadcast region. Alternatively, cluster trials of interventions targeted at the workplace to change culture and attitudes could be explored, similar to what was done in the Victorian Workcover back injury prevention programme [78].

In the short-term at least, clinical assessment of LBP, and assessment of treatment effect in trials, needs to move beyond pain and function to encompass the multidimensional impact on identity and social participation, discrimination, and worries about the future.

Georgy and colleagues in a review focusing on back pain patients’ and doctors’ expectations, found that patients and doctors conceptualise their expectations differently and have a wide-range of specific expectations for care [79]. Parsons and colleagues reported that trust, diagnosis, being believed, and good communication are important to chronic musculoskeletal pain patients [80]. Our findings are consistent with these reports but highlight areas on which health care providers may wish to focus in order to improve patient-experience. Using multidimensional scaling, Buchbinder and colleagues proposed a new model for assessing back pain outcomes [81]. Their model has six domains and 16 sub-domains, including several new domains not currently recommended: loss of independence, worry about the future, and negative or discriminatory actions by others. These were strong themes in our meta-analysis and we agree these warrant consideration in future measurement.

Sanders *et al.* provide a useful account contrasting the manual therapists’ perspectives of treating back pain patients [82]. In particular, they note that whilst there is recognition of the importance of a social and psychological approach to management, therapists felt that they did not possess adequate skills or training to deal with psychosocial obstacles effectively. Their analysis extends, and may further explain, an earlier interpretation from the same data that physical therapist’s approach to management is largely structural and mechanical [83]. We agree with Foster and Delitto that whilst there is considerable competition on undergraduate syllabus for biomedical modules, there may be an opportunity to improve and emphasise the integration of a biopsychosocial approach within entry-level training [84].

### Strengths and limitations of the study

Given the lack of consensus regarding the methodology of synthesis, it is encouraging that the results from the two independent approaches used by reviewers in this review were congruent. The different approaches were complementary for the purposes of synthesis; although arguably the rigour of future designs could be improved through independent but identical approaches. It is assumed that our included studies are commensurable. That is to say we considered it reasonable to synthesize results from these studies: all of the included studies involved people with non-specific LBP and all were studies of those people’s experiences. Commensurability may be likened to the discussion of heterogeneity in meta-analyses, which is considered both from clinical and statistical perspectives. In the absence of metrics in qualitative synthesis, judgment regarding commensurability must be made subjectively. We limited our review to face-to-face qualitative studies, excluding telephone interview studies, and quantitative studies. Whilst this helped with regards to commensurably, we risked excluding relevant information from mixed-methods and telephone-based studies. We did not identify any material associations between countries in which studies were conducted and descriptions of impact. However, as we focused on English language studies only, we acknowledge that this may have led to exclusion of geographical regions and therefore may have risked missing potentially unique associations by region. Only 10 of the 32 items from the consolidated criteria for reporting qualitative research framework, were judged as clearly documented by ≥ 70% of the included papers. This suggests that the comprehensiveness of the reporting of qualitative work in this field could be improved, although this may be independent of the core content of the studies, on which this review was based.

One might question whether the impact of chronic LBP is likely to differ from the impact of any other form of chronic pain; indeed over three-quarters of people with chronic pain have pain in multiple sites [85]. There are similarities between our findings and descriptions of the pervasive nature of chronic pain in other literature [86]. However, the experienced of those suffering from LBP, or ‘backnicks’ – a turn of phrase coined by Borkan *et al.* emphasising a perceived variant category of social role/expectation–[34] may materially differ from the experience of people with chronic musculoskeletal pain because of unique societal prejudice towards LBP sufferers.

## Conclusions

The development of next generation outcome measures for LBP should take social factors into account in addition to psychological and biological factors. However, a challenge remains in reaching consensus on what should be measured in trials, and which domains can reasonably be assessed by clinicians. We offer this review as material to inform and stimulate further debate. It is not clear whether current individual-targeted LBP interventions are able to act directly or in a timely fashion on such sociological impacts as stigma, or financial concerns. Neither is it clear, whether once psychological components have become established, alleviation of the pain component of LBP translates to concomitant alleviation of the associated mental health problems. Aiming to provide early tenable diagnosis, and proactive and holistic (*qua* comprehensive) treatment of back pain, whilst it is still a sub-acute phase, may lessen a long-term negative impact, avoid excessive costs and complications of secondary health effects. It may also improve the patient-practitioner relationship.

## Ethics approval

None required. However, the protocol for this review received favourable opinion from South East Coast Brighton & Sussex REC as part of a larger project (11-LO-1190).

## Competing interests

SP, CS, TP, DR, and CF declare that they have no conflicts of interest. RF and MU are also directors and shareholders of a company that provides electronic measurement services to health services researchers; notwithstanding this, they declare that they have no conflicts of interest.

## Authors’ contributions

RF conceived and led the study, coded data and developed the framework for first order coding, helped develop the final interpretation, abstracted and arbitrated quality data, was the first reviewer and wrote the first draft of the manuscript. SP was the second reviewer, helped develop the final interpretation, abstracted quality data, and commented on successive versions of the manuscript. SE contributed to study design, helped develop the final interpretation, and commented on successive drafts of the manuscript. CS helped to develop the final interpretation and commented on successive drafts of the manuscript, TP helped develop the coding framework, final interpretation, and commented on successive drafts of the manuscript. DR abstracted and arbitrated quality data, produced the figures. CF abstracted and arbitrated quality data from included studies. MU contributed to the study design, arbitrated studies for inclusion, helped develop the final interpretation, and commented on successive drafts of the manuscript. All authors discussed the results and commented on the manuscript, and all authors approved the final manuscript.

## Pre-publication history

The pre-publication history for this paper can be accessed here:

http://www.biomedcentral.com/1471-2474/15/50/prepub

## Supplementary Material

Additional file 1: Table S1 Search strategy details. Click here for file

Additional file 2: Table S2 Additional characteristics of included studies. Click here for file

Additional file 3 COREQ framework reporting criteria. Click here for file
