# Supplementary material for: A systematic review and meta-synthesis of the impact of low back pain on people’s lives
Source: BMC Musculoskelet Disord. 2014 Feb 21;15:50. doi: 10.1186/1471-2474-15-50 (PMC3932512; doi:10.1186/1471-2474-15-50)
Supplement: Additional file 1: Table S1 — Search strategy details. [file 1471-2474-15-50-S1.pdf]

Online

Table 1: Characteristics of included studies

Lead author and year	Sample size	% Female	Average* age (years)	Setting	Aim	Main Findings/conclusion
Allegretti, 2010	23	48	45	I	To explore patient and physician interviews and improve communication	Patients focus on suffering and convey reliance on physicians
Benjaminsson, 2007	17	59	36	I	To explore how patients respond to recurrence of pain	Patients respond differently, with variable motivation for change
Borkan, 1995	66	65	39.5	FG & I	To explore patients' perceptions and experiences	Patients describe a rich world of pain, are critical of the medical system, and choose treatment based on what they find works
Bowman, 1991	15	40	ND	I	To investigate the meaning of chronic LBP	The presence of pain affected every aspect of these subject's lives. Increased awareness of the impact of pain on individuals may lead to better management.
Bowman, 1994	15	40	ND	I	To describe life with LBP	LBP is multidimensional. Sufferers must deal with the uncertainty of the cause. Eventually, some accept the 'inevitable nature' of the pain
Bowman, 1994	15	40	ND	I	To examine the reaction of individuals to chronic LBP	Individuals experienced social isolation, enhanced awareness of others experiencing pain, and varied psychological reactions.
Bush	22	68	41	I	To examine the development of pain related appraisals, coping and well behaviours, as well as to investigate how these processes affect one another during the course of LBP	Acceptance of CLBP favoured rehabilitation and helped participants change pain-related behaviours. The majority of the participants used disregarding processes in response to CLBP. The disregarding process developed from a psychological defence into a conscious coping strategy, the transition mediated by a crisis. This defence seemed to protect the participants' self-concept and reduce emotional discomfort, although protect it did not promote rehabilitation. The disregarding strategy was usually employed in later phases of the disorder and was consistent with active attempts at changing pain-related behaviours
Campbell, 2007	16	ND	ND	FG	To examine expectations for pain treatment and outcome and to determine whether they are influential in maintaining health service consumption	Participants revealed considerable agency as they continued in a quest to engage and re-engage with the gatekeepers of health care facilities

Continued on next page...

Table 1 – Continued

Lead author and year	Sample size	% Female	Average* age (years)	Setting	Aim	Main Findings/conclusion
Chew, 1997	20	82	ND	I	To explore how sufferers of LBP describe their pain and its impact on their lives	Patients describe withdrawal from normal social obligations, including work. They view GPs as unable to help, leading GP to become a resource through which social and economic inactivity can be legitimated.
Cook, 2000	7	57	42.3	I	To gain an in-depth understanding of individual patients' experiences of LBP and active rehabilitation	Participants continued to express dependence on the health care professions
Coole, 2010	25	52	44.7	I	To explore the experiences of unemployed people with back pain regarding the help they have received from GPs	The perception of participants was that GPs and other clinicians had provided little or no work-focused guidance and support. Sickness certification was the main method used to manage participants' work problems
Coole, 2010b	25	52	44.7	I	To explore the individual experiences and perceptions of patients awaiting rehabilitation who were concerned about their ability to work because of persisting, or recurrent, low back pain	Workers were concerned about how back pain was viewed by their employers and co-workers and felt the need to justify their condition with a medical diagnosis and evidence
Corbett, 2007	37	59	ND	I	To explore the struggle between hope and despair through consideration of six people's narratives about their experiences of chronic LBP	Suffers must endure sufferer not only physical pain, but also the psychosocial implications, which pose an added and often complicated challenge.
Crowe, 2010	64	48	55.1	I	To investigate experiences of the impact of LBP	Pain is unpredictable, participants are vigilant, and externalization/ objectication of the body can alter the sense of self.
Dean, 2010	33	18	47.7	I	To explore and document the experiences of NZ farm workers who continue to work despite their LBP	This rural workforce adopts a 'can do' attitude to work, managing LBP within the context of having job control and flexible work practices
Hooper, 2005	2	50%	ND	I	to provide opportunities to reflect on clinical practice and on the role of informal carers within the provision of health care for the back pain patient	Analysis reveals a positive and mediating role for informal carers within the provision of health care
Holloway, 2007	18	50	53	I	To explore and conceptualise the experiences of people of working age who seek help from pain clinics for LBP	Stigmatisation emerged as a key theme from the narrative accounts of participants. The findings expose subtle as well as overt stigmatising responses by family, friends, health professionals and the general public which appeared to have a profound

Continued on next page...

Table 1 – Continued

Lead author and year	Sample size	% Female	Average* age (years)	Setting	Aim	Main Findings/conclusion
						effect on the perceptions, self esteem and behaviours
Hush, 2009	36	42	41	FG	To explore patients' perceptions of recovery from LBP	Recovery encompassed a range of factors that can be broadly classified into the domains of symptom attenuation, improved capacity to perform a broad scope of self-defined functional activities, and achievement of an acceptable quality of life
Hush, 2010b	36	42	41.6	FG	To explore whether NRS/RMDQ capture meaningful changes	Neither the NRS or RMDQ captures the complex personal experience of LBP
Keen, 1999	27	37	ND	I	To explore the association influence changes in physical activity and the way individuals perceive and behave with their LBP and the impact of this on physical activity	When advocating activity it is early as possible, and explore such issues when present
Layzell, 2001	12	50	ND	FG	To explore how back pain affects sufferers' lives	The issue of locus of control was a dominant theme throughout the study and those with stronger internal beliefs had a more positive outlook.
Liddle, 2007	28	78	ND	FG	To explore experiences, opinion, and treatment expectations of LBP to identify what treatment components are valued	Participants considered exercise and activity modification important and welcomed individually tailored advice and exercise programmes
May, 2000	12	50	ND	I	To explore ways persons with long standing chronic LBP respond to medical doubt about the presence of organic pathology	Patients stressed their own clinical expertise and suggested clinicians were confused or uncertain about diagnosis
Morris, 2004	6	50	ND	I	Programmes are designed to of change behaviour, increase activity levels and encourage self-management. Patients' experiences of attending a back rehabilitation programme were examined	The study demonstrated the importance of understanding patients' beliefs and expectations prior to a , back programme accounting for these during attendance and cultivate awareness of the impact of language
Ong, 2003	6	50	ND	FG	To describes course of LBP over 12 months	Tensions were revealed between patients and practitioners
Ong, 2004	16	38	ND	I	To explore how people report LBP to clinicians	Patients feel the need to be believable narrators in order to legitimise their pain
Ong, 2006	2	100	ND	I	To explore the role of concordance in therapeutic relationships through directly comparing patients' and clinicians' accounts of the diagnosis and impact of LBP	Patient/Health Practitioner concordance/discordance, is unrelated to cost effectiveness or clinical effectiveness
Osborn,	9	100	ND	I	To explore the sufferer's	Patients felt unable to explain the

Continued on next page. . .

Table 1 – Continued

Lead author and year	Sample size	% Female	Average* age (years)	Setting	Aim	Main Findings/conclusion
1998					personal experiences of their pain	persistence of their pain, and felt stigmatised, leading to withdrawal from social contact
Osborn, 2006	6	40	44	I	To explore and articulate the meanings and themes that make up the personal experience of the body when in pain	The embodied unpleasantness of chronic pain involves an assault upon and a defence of a preferred or desirable self
Reid, 2004	50	54	ND	I	To explore the perceived health needs of chronic LBP patients	A patient-centred procedure could be helpful in outlining individual concerns, translatable into recovery goals for intervention, regardless of concurrent medical treatment
Skelton, 1996	52	50	41	I	To elicit the views of patients concerning LBP and its management in general practice	Patients' views on LBP are heterogeneous. Dissatisfaction expressed with medical explanations may be related to superficial clinical management and the constraints of general practice
Slade, 2009	18	50	51.2	FG	To determine participant experience of exercise programs for non-specific LBP	The ramifications of stigma and discrimination are enduring, potentially disabling and appear to interfere with care-seeking, rehabilitation participation, and potentially, rehabilitation outcomes
Slade, 2009	18	50	51	FG	To determine what factors are important for patients to engage in exercise programmes	People are more likely to participate if their preferences, circumstances and past exercise experiences are considered
Slade, 2009	18	50	51	FG	To investigate and summarise participant experience of exercise programmes for non-specific LBP and the effects of these experiences on exercise participation and engagement	A gap exists between care-seeker experiences of, and preferences for, exercise programmes for back pain
Sloots, 2010	23	52	40	I	To explore which factors led to drop-out in patients of Turkish and Moroccan origin with chronic nonspecific LBP who participated in a rehabilitation programme	The major reason for drop-out was patients having different expectations, from those of their health providers, of the aim of treatment, as a result of a different view of the origin and treatment of LBP
Smith, 2007	6	33	44	I	To explore how chronic benign low back pain may have a serious debilitating impact on the sufferer's sense of self	Chronic pain impacts of patients self and identity, especially in a public arena
Snelgrove, 2009	10	70	ND	I	To understand the meaning of LBP for participants with longstanding history of chronic pain	Patients frame experience in biomedical model may thus limit their management to medical only interventions
de Souza, 2007	11	55	49.3	I	To explore and describe the physical consequences of living day-to-day with LBP	Participants expressed regret at the loss of capabilities and distress at the functional consequences of

Continued on next page. . .

Table 1 – Continued

Lead author and year	Sample size	% Female	Average* age (years)	Setting	Aim	Main Findings/conclusion
					and to document insider accounts of how the pain impacts daily activities	those losses. Main themes were: sleep/rest, mobility, independence and leisure
de Souza, 2011	11	55	49.3	I	To explore interactions and relationships within the family and the workplace from the perspective of the person with chronic spinal pain	Patients valued support from family but expressed concerns about causing them worry, Work-related issues included physical and emotional efforts to keep working when in pain, fear of losing employment and financial problems.
Sokunbi, 2010	9	67	46.6	FG	To explore the experiences of a sample of individuals with chronic LBP who participated in an RCT of exercises	Experiences included the positive effects of stabilisation exercises on pain, functional disability and quality of life, and also reflected increases in confidence, the formulation of self help strategies and the ability to exert better control over their LBP
Strong, 1995	19	58	53.7	FG	To explore coping strategies	A somatic focus was adopted with reliance on planful action and variation.
Strong, 1994	8	50	54.4	FG	To explore relevant dimensions of pain	Relationships, negative affects emotions, symptoms, features mobility, domestic activities and treatment were important
Tarasuk, 1995	15	33	ND	I	To learn about individuals' experiences and perspectives of longer term ramifications of LBP	Patients felt that their legitimacy was in doubt within the workplace despite having their problem 'legitimised' by medical and compensatory profession
Tavafian, 2008	24	100	42.9	FG	To explore Iranian womens' beliefs about causation	Participants felt LBP was a consequence of stress, meeting expectations, and housework.
Tveito 1995	15	33	ND	I	To address legitimacy concerns in the workplace, particularly those relating to workers' perceptions of reactions of employers, supervisors, and co-workers	Injured workers encountered suspicion in their workplaces regarding the validity of their claims to back injury and their need for compensable time off work; this was associated with damaged relations and perceived job insecurity
Young, 2011	31	45	ND	FG	To determine the meaning participants associated with the term 'recurrence'	Participants described their experiences in a way that is consistent with the idea that LBP is a fluctuating and disabling health condition. Results cast doubt on the validity of currently available measures of LBP recurrence.
Wade, 2003	3	100	ND	I	To provide a description of the life-world of people with chronic low back pain	Chronic pain causes a disruption in the relationship between the person and the body; sufferers are unable to fulfil social roles as expected and are forced to revise their goals and

Continued on next page. . .

Table 1 – Continued

Lead author and year	Sample size	% Female	Average* age (years)	Setting	Aim	Main Findings/conclusion
						activities to accommodate the pain. The distress of their condition is mediated by the ability to make sense of their condition, finding meaning in the pain
Walker, 1999	20	40	ND	I	ND	Participants became entrapped within the medical, social security and legal systems. These systems, designed to treat or support those who are ill or disabled, effectively rendered participants powerless, helpless and angry
Walker, 2006	20	40	ND	I	To provide a more detailed understanding of the lived experience of chronic back pain prior to seeking help from pain clinics	Material losses, as well as perceptions of loss, are prominent issues for those of working age seeking help from pain clinics for chronic back pain, and may need to be acknowledged and addressed as part of therapeutic interventions. The possibility of age-related differences in pain-related loss may be worthy of further investigation

* Mean or median age; FG - Focus Group; I - Interviews
