# Supplementary material for: A systematic review and meta-synthesis of the impact of low back pain on people’s lives
Source: BMC Musculoskelet Disord. 2014 Feb 21;15:50. doi: 10.1186/1471-2474-15-50 (PMC3932512; doi:10.1186/1471-2474-15-50)
Supplement: Additional file 2: Table S2 — Additional characteristics of included studies. [file 1471-2474-15-50-S2.pdf]

## Online

Table 1: Additional characteristics of included studies

| Lead author and year | Described analytical framework | Setting | Aim | Main Findings/conclusion |
|---|---|---|---|---|
| Allegretti, 2010 | Immersion-crystalisation | I | To explore patient and physician interviews and improve communication | Patients focus on suffering and convey reliance on physicians |
| Benjaminsson, 2007 | Phenomenology | I | To explore how patients respond to recurrence of pain | Patients respond differently, with variable motivation for change |
| Borkan, 1995 | Immersion-crystalisation | FG & I | To explore patients' perceptions and experiences | Patients describe a rich world of pain, are critical of the medical system, and choose treatment based on what they find works |
| Bowman, 1991 | Phenomenology (Parse, 1985) | I | To investigate the meaning of chronic LBP | The presence of pain affected every aspect of these subject's lives. Increased awareness of the impact of pain on individuals may lead to better management. |
| Bowman, 1994 | Frames of reference | I | To describe life with LBP | LBP is multidimensional. Sufferers must deal with the uncertainty of the cause. Eventually, some accept the 'inevitable nature' of the pain |
| Bowman, 1994 | ND | I | To examine the reaction of individuals to chronic LBP | Individuals experienced social isolation, enhanced awareness of others experiencing pain, and varied psychological reactions. |
| Bush, 2005 | Grounded Theory | I | To examine the development of pain related appraisals, coping and well behaviours, as well as to investigate how these processes affect one another during the course of LBP | Acceptance of CLBP favoured rehabilitation and helped participants change pain-related behaviours. The majority of the participants used disregarding processes in response to CLBP. The disregarding process developed from a psychological defence into a conscious coping strategy, the transition mediated by a crisis. This defence seemed to protect the participants' self-concept and reduce emotional discomfort, although protect it did not promote rehabilitation. The disregarding strategy was usually employed in later phases of the disorder and was consistent with active attempts at changing pain-related behaviours |
| Campbell, 2007 | Thematic extraction | FG | To examine expectations for pain treatment and outcome and to determine whether they are influential in maintaining | Participants revealed considerable agency as they continued in a quest to engage and re-engage with the gatekeepers of health care facilities |

Continued on next page...

Table 1 – Continued

| Lead author and year | Described analytical framework | Setting | Aim | Main Findings/conclusion |
|---|---|---|---|---|
| | | | health service consumption | |
| Chew, 1997 | Conventional (Strauss, 1987) | I | To explore how sufferers of LBP describe their pain and its impact on their lives | Patients describe withdrawal from normal social obligations, including work. They view GPs as unable to help, leading GP to become a resource through which social and economic inactivity can be legitimated. |
| Cook, 2000 | Constant comparative method (Glaser & Strauss, 1967) | I | To gain an in-depth understanding of individual patients' experiences of LBP and active rehabilitation | Participants continued to express dependence on the health care professions |
| Coole, 2010 | Thematic extraction | I | To explore the experiences of employed people with back pain regarding the help they have received from GPs | The perception of participants was that GPs and other clinicians had provided little or no work-focused guidance and support. Sickness certification was the main method used to manage participants' work problems |
| Coole, 2010b | Thematic analysis | I | To explore the individual experiences and perceptions of patients awaiting rehabilitation who were concerned about their ability to work because of persisting, or recurrent, low back pain | Workers were concerned about how back pain was viewed by their employers and co-workers and felt the need to justify their condition with a medical diagnosis and evidence |
| Corbett, 2007 | Thematic analysis | I | To explore the struggle between hope and despair through consideration of six people's narratives about their experiences of chronic LBP | Suffers must endure sufferer not only physical pain, but also the psychosocial implications, which pose an added and often complicated challenge. |
| Crowe, 2010 | Thematic analysis | I | To investigate experiences of the impact of LBP | Pain is unpredictable, participants are vigilant, and externalization/ objectication of the body can alter the sense of self. |
| Dean, 2010 | IPA | I | To explore and document the experiences of NZ farm workers who continue to work despite their LBP | This rural workforce adopts a 'can do' attitude to work, managing LBP within the context of having job control and flexible work practices |
| Hooper, 2005 | Thematic/narrative analysis | I | To provide opportunities to reflect on clinical practice and on the role of informal carers within the provision of health care for the back pain patient | Analysis reveals a positive and mediating role for informal carers within the provision of health care |
| Holloway, 2007 | IPA | I | To explore and conceptualise the experiences of people of working age who seek help from pain clinics for LBP | Stigmatisation emerged as a key theme from the narrative accounts of participants. The findings expose subtle as well as overt stigmatising responses by family, friends, health |

Continued on next page. . .

Table 1 – Continued

| Lead author and year | Described analytical framework | Setting | Aim | Main Findings/conclusion |
|---|---|---|---|---|
| | | | | professionals and the general public which appeared to have a profound effect on the perceptions, self esteem and behaviours |
| Hush, 2009 | Framework | FG | To explore patients' perceptions of recovery from LBP | Recovery encompassed a range of factors that can be broadly classified into the domains of symptom attenuation, improved capacity to perform a broad scope of self-defined functional activities, and achievement of an acceptable quality of life |
| Hush, 2010b | Framework | FG | To explore whether NRS/RMDQ capture meaningful changes | Neither the NRS or RMDQ captures the complex personal experience of LBP |
| Keen, 1999 | Framework | I | To explore the association influence changes in physical activity and the way individuals perceive and behave with their LBP and the impact of this on physical activity | When advocating activity it is early as possible, and explore such issues when present |
| Layzell, 2001 | Emerging themes | FG | To explore how back pain affects sufferers' lives | The issue of locus of control was a dominant theme throughout the study and those with stronger internal beliefs had a more positive outlook. |
| Liddle, 2007 | Thematic extraction | FG | To explore experiences, opinion, and treatment expectations of LBP to identify what treatment components are valued | Participants considered exercise and activity modification important and welcomed individually tailored advice and exercise programmes |
| May, 2000 | Conventional model of content analysis (Strauss) | I | To explore ways persons with long standing chronic LBP respond to medical doubt about the presence of organic pathology | Patients stressed their own clinical expertise and suggested clinicians were confused or uncertain about diagnosis |
| Morris, 2004 | Thematic content analysis | I | Programmes are designed to of change behaviour, increase activity levels and encourage self-management. Patients' experiences of attending a back rehabilitation programme were examined | The study demonstrated the importance of understanding patients' beliefs and expectations prior to a , back programme accounting for these during attendance and cultivate awareness of the impact of language |
| Ong, 2003 | Thematic analysis | FG | To describes course of LBP over 12 months | Tensions were revealed between patients and practitioners |
| Ong, 2004 | Grounded theory | I | To explore how people report LBP to clinicians | Patients feel the need to be believable narrators in order to legitimise their pain |
| Ong, 2006 | Grounded theory | I | To explore the role of concordance in therapeutic relationships through directly comparing patients' and clinicians' accounts of the | Patient/Health Practitioner concordance/discordance, is unrelated to cost effectiveness or clinical effectiveness |

Continued on next page...

Table 1 – Continued

| Lead author and year | Described analytical framework | Setting | Aim | Main Findings/conclusion |
|---|---|---|---|---|
| | | | diagnosis and impact of LBP | |
| Osborn, 1998 | IPA | I | To explore the sufferer's personal experiences of their pain | Patients felt unable to explain the persistence of their pain, and felt stigmatised, leading to withdrawal from social contact |
| Osborn, 2006 | IPA | I | To explore and articulate the meanings and themes that make up the personal experience of the body when in pain | The embodied unpleasantness of chronic pain involves an assault upon and a defence of a preferred or desirable self |
| Reid, 2004 | Thematic extraction | I | To explore the perceived health needs of chronic LBP patients | A patient-centred procedure could be helpful in outlining individual concerns, translatable into recovery goals for intervention, regardless of concurrent medical treatment |
| Skelton, 1996 | Thematic extraction | I | To elicit the views of patients concerning LBP and its management in general practice | Patients' views on LBP are heterogeneous. Dissatisfaction expressed with medical explanations may be related to superficial clinical management and the constraints of general practice |
| Slade, 2009 | Grounded theory | FG | To determine participant experience of exercise programs for non-specific LBP | The ramifications of stigma and discrimination are enduring, potentially disabling and appear to interfere with care-seeking, rehabilitation participation, and potentially, rehabilitation outcomes |
| Slade, 2009 | Grounded theory | FG | To determine what factors are important for patients to engage in exercise programmes | People are more likely to participate if their preferences, circumstances and past exercise experiences are considered |
| Slade, 2009 | Grounded theory | FG | To investigate and summarise participant experience of exercise programmes for non-specific LBP and the effects of these experiences on exercise participation and engagement | A gap exists between care-seeker experiences of, and preferences for, exercise programmes for back pain |
| Sloots, 2010 | Thematic extraction | I | To explore which factors led to drop-out in patients of Turkish and Moroccan origin with chronic nonspecific LBP who participated in a rehabilitation programme | The major reason for drop-out was patients having different expectations, from those of their health providers, of the aim of treatment, as a result of a different view of the origin and treatment of LBP |
| Smith, 2007 | IPA | I | To explore how chronic benign low back pain may have a serious debilitating impact on the sufferer's sense of self | Chronic pain impacts of patients self and identity, especially in a public arena |
| Snelgrove, 2009 | Thematic extraction | I | To understand the meaning of LBP for participants with longstanding history of chronic pain | Patients frame experience in biomedical model may thus limit their management to medical only interventions |

Continued on next page...

Table 1 – Continued

| Lead author and year | Described analytical framework | Setting | Aim | Main Findings/conclusion |
|---|---|---|---|---|
| de Souza, 2007 | Framework | I | To explore and describe the physical consequences of living day-to-day with LBP and to document insider accounts of how the pain impacts daily activities | Participants expressed regret at the loss of capabilities and distress at the functional consequences of those losses. Main themes were: sleep/rest, mobility, independence and leisure |
| de Souza, 2011 | Framework | I | To explore interactions and relationships within the family and the workplace from the perspective of the person with chronic spinal pain | Patients valued support from family but expressed concerns about causing them worry, Work-related issues included physical and emotional efforts to keep working when in pain, fear of losing employment and financial problems. |
| Sokunbi, 2010 | Thematic analysis | FG | To explore the experiences of a sample of individuals with chronic LBP who participated in an RCT of exercises | Experiences included the positive effects of stabilisation exercises on pain, functional disability and quality of life, and also reflected increases in confidence, the formulation of self help strategies and the ability to exert better control over their LBP |
| Strong, 1995 | Content analysis with thematic abstraction | FG | To explore coping strategies | A somatic focus was adopted with reliance on planful action and variation. |
| Strong, 1994 | Content analysis with frequency count of themes | FG | To explore relevant dimensions of pain | Relationships, negative affects emotions, symptoms, features mobility, domestic activities and treatment were important |
| Tarasuk, 1995 | Grounded theory | I | To learn about individuals' experiences and perspectives of longer term ramifications of LBP | Patients felt that their legitimacy was in doubt within the workplace despite having their problem 'legitimised' by medical and compensatory profession |
| Tavafian, 2008 | Content analysis with open coding and thematic extraction | FG | To explore Iranian womens' beliefs about causation | Participants felt LBP was a consequence of stress, meeting expectations, and housework. |
| Tveito 1995 | Thematic extraction and grounded theory | I | To address legitimacy concerns in the workplace, particularly those relating to workers' perceptions of reactions of employers, supervisors, and co-workers | Injured workers encountered suspicion in their workplaces regarding the validity of their claims to back injury and their need for compensable time off work; this was associated with damaged relations and perceived job insecurity |
| Young, 2011 | Use of ICF as coding framework | FG | To determine the meaning participants associated with the term 'recurrence' | Participants described their experiences in a way that is consistent with the idea that LBP is a fluctuating and disabling health condition. Results cast doubt on the validity of currently available measures of LBP recurrence. |
| Wade, | Phenomenology | I | To provide a description of | Chronic pain causes a disruption in |

Continued on next page. . .

Table 1 – Continued

| Lead author and year | Described analytical framework | Setting | Aim | Main Findings/conclusion |
|---|---|---|---|---|
| 2003 | (Kruger, 1979) | | the life-world of people with chronic low back pain | the relationship between the person and the body; sufferers are unable to fulfil social roles as expected and are forced to revise their goals and activities to accommodate the pain. The distress of their condition is mediated by the ability to make sense of their condition, finding meaning in the pain |
| Walker, 1999 | IPA | I | ND | Participants became entrapped within the medical, social security and legal systems. These systems, designed to treat or support those who are ill or disabled, effectively rendered participants powerless, helpless and angry |
| Walker, 2006 | Phenomenology (Smith, 1996) | I | To provide a more detailed understanding of the lived experience of chronic back pain prior to seeking help from pain clinics | Material losses, as well as perceptions of loss, are prominent issues for those of working age seeking help from pain clinics for chronic back pain, and may need to be acknowledged and addressed as part of therapeutic interventions. The possibility of age-related differences in pain-related loss may be worthy of further investigation |

* Mean or median age; FG - Focus Group; I - Interviews; IPA = Interpretive Phenomenological Analysis
