# Supplementary material for: A systematic review and meta-synthesis of the impact of low back pain on people’s lives
Source: BMC Musculoskelet Disord. 2014 Feb 21;15:50. doi: 10.1186/1471-2474-15-50 (PMC3932512; doi:10.1186/1471-2474-15-50)
Supplement: Additional file 3 — COREQ framework reporting criteria. [file 1471-2474-15-50-S3.docx]

**Additional file 3: COREQ framework reporting criteria**

| **Reporting criterion** | **No(%), n=49** | **References for studies reporting criterion** |
| --- | --- | --- |
| *Characteristics of research team:*  Interviewer or facilitator  Researcher’s credentials  Researcher’s occupation  Researcher’s gender  Interviewer/facilitator experience and training | **29 (59)**  **13 (27)**  **16 (33)**  **6 (12)**  **8 (16)** | **[**[**27**](#_ENREF_27)**,** [**28**](#_ENREF_28)**,** [**31**](#_ENREF_31)**,** [**33-36**](#_ENREF_33)**,** [**38**](#_ENREF_38)**,** [**39**](#_ENREF_39)**,** [**41**](#_ENREF_41)**,** [**43**](#_ENREF_43)**,** [**45-47**](#_ENREF_45)**,** [**50-54**](#_ENREF_50)**,** [**57**](#_ENREF_57)**,** [**58**](#_ENREF_58)**,** [**60**](#_ENREF_60)**,** [**64-67**](#_ENREF_64)**,** [**69**](#_ENREF_69)**,** [**71**](#_ENREF_71)**,** [**73**](#_ENREF_73)**]**  **[**[**29**](#_ENREF_29)**,** [**35-38**](#_ENREF_35)**,** [**41**](#_ENREF_41)**,** [**53**](#_ENREF_53)**,** [**58**](#_ENREF_58)**,** [**61**](#_ENREF_61)**,** [**62**](#_ENREF_62)**,** [**66**](#_ENREF_66)**,** [**67**](#_ENREF_67)**,** [**70**](#_ENREF_70)**]**  **[**[**12**](#_ENREF_12)**,** [**28**](#_ENREF_28)**,** [**30**](#_ENREF_30)**,** [**33**](#_ENREF_33)**,** [**38**](#_ENREF_38)**,** [**39**](#_ENREF_39)**,** [**41**](#_ENREF_41)**,** [**43**](#_ENREF_43)**,** [**47**](#_ENREF_47)**,** [**51**](#_ENREF_51)**,** [**57**](#_ENREF_57)**,** [**58**](#_ENREF_58)**,** [**64**](#_ENREF_64)**,** [**66**](#_ENREF_66)**,** [**69**](#_ENREF_69)**,** [**72**](#_ENREF_72)**]**  **[**[**13**](#_ENREF_13)**,** [**30**](#_ENREF_30)**,** [**39**](#_ENREF_39)**,** [**41**](#_ENREF_41)**,** [**62**](#_ENREF_62)**,** [**66**](#_ENREF_66)**]**  **[**[**41**](#_ENREF_41)**,** [**44**](#_ENREF_44)**,** [**51**](#_ENREF_51)**,** [**53**](#_ENREF_53)**,** [**57**](#_ENREF_57)**,** [**59**](#_ENREF_59)**,** [**61**](#_ENREF_61)**,** [**71**](#_ENREF_71)**]** |
| *Relationship with participants:*  Relationship established  Knowledge of interviewer‘s background | **2 (4)**  **0 (0)** | **[**[**53**](#_ENREF_53)**,** [**66**](#_ENREF_66)**]** |
| *Methodological theory identified* | **49(100)** | **[**[**12**](#_ENREF_12)**,** [**13**](#_ENREF_13)**,** [**27-73**](#_ENREF_27)**]** |
|  |  |  |
| *Participant selection:* |  |  |
| Sampling method  Method of approach  Sample size  Refusals or non-participants | **47 (96)**  **28 (57)**  **49 (100)**  **23 (47)** | **[**[**12**](#_ENREF_12)**,** [**13**](#_ENREF_13)**,** [**27-40**](#_ENREF_27)**,** [**42-46**](#_ENREF_42)**,** [**48-73**](#_ENREF_48)**]**  **[**[**12**](#_ENREF_12)**,** [**27**](#_ENREF_27)**,** [**31-33**](#_ENREF_31)**,** [**35-38**](#_ENREF_35)**,** [**42-44**](#_ENREF_42)**,** [**50-53**](#_ENREF_50)**,** [**57-61**](#_ENREF_57)**,** [**63**](#_ENREF_63)**,** [**64**](#_ENREF_64)**,** [**66-69**](#_ENREF_66)**,** [**73**](#_ENREF_73)**]**  **[**[**12**](#_ENREF_12)**,** [**13**](#_ENREF_13)**,** [**27-73**](#_ENREF_27)**]**  **[**[**27**](#_ENREF_27)**,** [**32**](#_ENREF_32)**,** [**34**](#_ENREF_34)**,** [**38**](#_ENREF_38)**,** [**39**](#_ENREF_39)**,** [**42**](#_ENREF_42)**,** [**45**](#_ENREF_45)**,** [**46**](#_ENREF_46)**,** [**50**](#_ENREF_50)**,** [**52**](#_ENREF_52)**,** [**53**](#_ENREF_53)**,** [**57-59**](#_ENREF_57)**,** [**61**](#_ENREF_61)**,** [**63-67**](#_ENREF_63)**,** [**69**](#_ENREF_69)**,** [**71**](#_ENREF_71)**,** [**72**](#_ENREF_72)**]** |
| *Setting:*  Setting of data collection  Presence of non-participants  Description of sample* | **26 (53)**  **13 (27)**  **34 (69)** | **[**[**12**](#_ENREF_12)**,** [**27**](#_ENREF_27)**,** [**29**](#_ENREF_29)**,** [**31-34**](#_ENREF_31)**,** [**36**](#_ENREF_36)**,** [**38**](#_ENREF_38)**,** [**41-43**](#_ENREF_41)**,** [**45**](#_ENREF_45)**,** [**46**](#_ENREF_46)**,** [**48**](#_ENREF_48)**,** [**51**](#_ENREF_51)**,** [**53**](#_ENREF_53)**,** [**54**](#_ENREF_54)**,** [**62**](#_ENREF_62)**,** [**64**](#_ENREF_64)**,** [**65**](#_ENREF_65)**,** [**67**](#_ENREF_67)**,** [**69**](#_ENREF_69)**,** [**71-73**](#_ENREF_71)**]**  **[**[**28**](#_ENREF_28)**,** [**29**](#_ENREF_29)**,** [**39**](#_ENREF_39)**,** [**51**](#_ENREF_51)**,** [**53**](#_ENREF_53)**,** [**54**](#_ENREF_54)**,** [**62**](#_ENREF_62)**,** [**63**](#_ENREF_63)**,** [**67-69**](#_ENREF_67)**,** [**71**](#_ENREF_71)**,** [**73**](#_ENREF_73)**]**  **[**[**12**](#_ENREF_12)**,** [**13**](#_ENREF_13)**,** [**27**](#_ENREF_27)**,** [**28**](#_ENREF_28)**,** [**30**](#_ENREF_30)**,** [**32-34**](#_ENREF_32)**,** [**41**](#_ENREF_41)**,** [**42**](#_ENREF_42)**,** [**44-48**](#_ENREF_44)**,** [**51-53**](#_ENREF_51)**,** [**56-69**](#_ENREF_56)**,** [**72**](#_ENREF_72)**,** [**73**](#_ENREF_73)**]** |
| *Data collection:*  Interview guide used  Repeat interviews conducted  Audio or visual recording  Field notes  Duration  Data saturation discussed  Transcripts returned to participants | **30 (61)**  **3 (6)**  **47 (96)**  **17 (35)**  **34 (69)**  **19 (39)**  **6 (12)** | **[**[**12**](#_ENREF_12)**,** [**27**](#_ENREF_27)**,** [**31-34**](#_ENREF_31)**,** [**38**](#_ENREF_38)**,** [**41-48**](#_ENREF_41)**,** [**50**](#_ENREF_50)**,** [**51**](#_ENREF_51)**,** [**53**](#_ENREF_53)**,** [**55**](#_ENREF_55)**,** [**56**](#_ENREF_56)**,** [**59-64**](#_ENREF_59)**,** [**67-69**](#_ENREF_67)**,** [**73**](#_ENREF_73)**]**  **[**[**27**](#_ENREF_27)**,** [**30**](#_ENREF_30)**,** [**39**](#_ENREF_39)**]**  **[**[**12**](#_ENREF_12)**,** [**13**](#_ENREF_13)**,** [**27-48**](#_ENREF_27)**,** [**50-56**](#_ENREF_50)**,** [**58-73**](#_ENREF_58)**]**  **[**[**12**](#_ENREF_12)**,** [**13**](#_ENREF_13)**,** [**28**](#_ENREF_28)**,** [**32**](#_ENREF_32)**,** [**39**](#_ENREF_39)**,** [**42**](#_ENREF_42)**,** [**48**](#_ENREF_48)**,** [**51**](#_ENREF_51)**,** [**53**](#_ENREF_53)**,** [**54**](#_ENREF_54)**,** [**60-62**](#_ENREF_60)**,** [**67-69**](#_ENREF_67)**,** [**72**](#_ENREF_72)**]**  **[**[**12**](#_ENREF_12)**,** [**13**](#_ENREF_13)**,** [**27**](#_ENREF_27)**,** [**31-39**](#_ENREF_31)**,** [**41**](#_ENREF_41)**,** [**42**](#_ENREF_42)**,** [**45**](#_ENREF_45)**,** [**46**](#_ENREF_46)**,** [**48**](#_ENREF_48)**,** [**51-53**](#_ENREF_51)**,** [**57-62**](#_ENREF_57)**,** [**64**](#_ENREF_64)**,** [**66-69**](#_ENREF_66)**,** [**71-73**](#_ENREF_71)**]**  **[**[**13**](#_ENREF_13)**,** [**27**](#_ENREF_27)**,** [**32**](#_ENREF_32)**,** [**34-36**](#_ENREF_34)**,** [**38**](#_ENREF_38)**,** [**39**](#_ENREF_39)**,** [**51**](#_ENREF_51)**,** [**53**](#_ENREF_53)**,** [**59-62**](#_ENREF_59)**,** [**64**](#_ENREF_64)**,** [**66**](#_ENREF_66)**,** [**67**](#_ENREF_67)**,** [**70**](#_ENREF_70)**,** [**73**](#_ENREF_73)**]**  **[**[**49**](#_ENREF_49)**,** [**51**](#_ENREF_51)**,** [**53**](#_ENREF_53)**,** [**54**](#_ENREF_54)**,** [**65**](#_ENREF_65)**,** [**67**](#_ENREF_67)**]** |
| *Data analysis:*  Number of data coders  Description of coding tree  Derivation of themes  Use of software  Participants’ feedback or member checking | **39 (80)**  **9 (18)**  **48 (98)**  **10 (20)**  **5 (10)** | **[**[**13**](#_ENREF_13)**,** [**27-29**](#_ENREF_27)**,** [**31-34**](#_ENREF_31)**,** [**38**](#_ENREF_38)**,** [**39**](#_ENREF_39)**,** [**41-48**](#_ENREF_41)**,** [**51-57**](#_ENREF_51)**,** [**59-67**](#_ENREF_59)**,** [**69-73**](#_ENREF_69)**]**  **[**[**12**](#_ENREF_12)**,** [**32**](#_ENREF_32)**,** [**41**](#_ENREF_41)**,** [**44**](#_ENREF_44)**,** [**66**](#_ENREF_66)**,** [**68**](#_ENREF_68)**,** [**69**](#_ENREF_69)**,** [**71**](#_ENREF_71)**,** [**72**](#_ENREF_72)**]**  **[**[**12**](#_ENREF_12)**,** [**13**](#_ENREF_13)**,** [**27-39**](#_ENREF_27)**,** [**41-73**](#_ENREF_41)**]**  **[**[**27**](#_ENREF_27)**,** [**30**](#_ENREF_30)**,** [**31**](#_ENREF_31)**,** [**39**](#_ENREF_39)**,** [**42**](#_ENREF_42)**,** [**43**](#_ENREF_43)**,** [**47**](#_ENREF_47)**,** [**51**](#_ENREF_51)**,** [**62**](#_ENREF_62)**,** [**66**](#_ENREF_66)**]**  **[**[**39**](#_ENREF_39)**,** [**41**](#_ENREF_41)**,** [**51**](#_ENREF_51)**,** [**62**](#_ENREF_62)**,** [**65**](#_ENREF_65)**]** |
| *Reporting:*  Participant quotations provided  Data and findings consistent  Clarity of major themes  Clarity of minor themes | **49 (100)**  **49 (100)**  **47 (96)**  **38 (78)** | **[**[**12**](#_ENREF_12)**,** [**13**](#_ENREF_13)**,** [**27-73**](#_ENREF_27)**]**  **[**[**12**](#_ENREF_12)**,** [**13**](#_ENREF_13)**,** [**27-73**](#_ENREF_27)**]**  **[**[**12**](#_ENREF_12)**,** [**13**](#_ENREF_13)**,** [**27-31**](#_ENREF_27)**,** [**33-39**](#_ENREF_33)**,** [**41-73**](#_ENREF_41)**]**  **[**[**12**](#_ENREF_12)**,** [**13**](#_ENREF_13)**,** [**30**](#_ENREF_30)**,** [**31**](#_ENREF_31)**,** [**33-35**](#_ENREF_33)**,** [**37-51**](#_ENREF_37)**,** [**57-64**](#_ENREF_57)**,** [**66-73**](#_ENREF_66)**]** |
